# MSC-secreted TGF-β regulates lipopolysaccharide-stimulated macrophage M2-like polarization via the Akt/FoxO1 pathway

**DOI:** 10.1186/s13287-019-1447-y

**Published:** 2019-11-26

**Authors:** Feng Liu, Haibo Qiu, Ming Xue, Shi Zhang, Xiwen Zhang, Jingyuan Xu, Jianxiao Chen, Yi Yang, Jianfeng Xie

**Affiliations:** Department of Critical Care Medicine, Zhongda Hospital, School of Medicine, Southeast University, No.87, Dingjiaqiao, Gulou District, Nanjing, 210009 China

**Keywords:** Sepsis, Transforming growth factor beta, Mesenchymal stem cells, Macrophages, Akt/FoxO1

## Abstract

**Background:**

An uncontrolled inflammatory response is a critical pathophysiological feature of sepsis. Mesenchymal stem cells (MSCs) induce macrophage phenotype polarization and reduce inflammation in sepsis. MSC-secreted transforming growth factor beta (TGF-β) participated in the immune modulatory function of MSCs. However, the underlying mechanism of MSC-secreted TGF-β was not fully elucidated in regulation macrophage M2-like polarization.

**Methods:**

The paracrine effects of MSCs on macrophage polarization were studied using a co-culture protocol with LPS-stimulated RAW264.7 cells/mouse peritoneal macrophages and MSCs. The effect of TGF-β in the co-culture system was blocked by the TGF-β receptor inhibitor. To determine the role of MSC-secreted TGF-β, we used recombinant TGF-β to culture with LPS-stimulated RAW264.7 cells. In addition, we employed antibody microarray analysis to determine the mechanisms of MSC secreted TGF-β on LPS-stimulated RAW264.7 cell/mouse peritoneal macrophage M2-like polarization. Furthermore, we used an Akt inhibitor and a FoxO1 inhibitor to inhibit the Akt/FoxO1 pathway. The nuclear translocation of FoxO1 was detected by Western blot.

**Results:**

MSCs induced LPS-stimulated RAW264.7 cell/mouse peritoneal macrophage polarization towards the M2-like phenotype and significantly reduced pro-inflammatory cytokine levels via paracrine, which was inhibited by TGF-β receptor inhibitor. Furthermore, we found that MSC-secreted TGF-β enhanced the macrophage phagocytic ability. The antibody microarray analysis and Western blot verified that TGF-β treatment activated the Akt/FoxO1 pathway in LPS-stimulated macrophages, TGF-β-induced FoxO1 nuclear translocation and obviously expressed in the cytoplasm, the effects of TGF-β regulatory effects on LPS-stimulated macrophage were inhibited by pre-treatment with Akt inhibitor and FoxO1 inhibitor.

**Conclusions:**

TGF-β secreted by MSCs could skew LPS-stimulated macrophage polarization towards the M2-like phenotype, reduce inflammatory reactions, and improve the phagocytic ability via the Akt/FoxO1 pathway, providing potential therapeutic strategies for sepsis.

## Background

Sepsis is defined as life-threatening organ dysfunction caused by a disordered host response to infection [1], and the mortality is as high as 30–50% [2, 3]. An uncontrolled inflammatory response that is induced by pathogens and the subsequent organ dysfunction characteristic of sepsis [4]. Hence, exploring new therapies to resolve this excessive and uncontrolled inflammatory response in sepsis is an active research topic.

Mesenchymal stem cells (MSCs) have immunomodulatory abilities, making these cells a novel cell-based therapeutic tool for sepsis [5]. A number of studies have shown that MSCs improve survival in experimental models of sepsis by modulating the uncontrolled inflammatory response against bacteria by reprogramming the macrophage phenotype [6, 7]. Therefore, MSCs that regulate the macrophage phenotype are critical for treating sepsis.

Macrophages are a key component in innate immunity and play a vital role in homeostasis and inflammatory diseases [8]. Based on environmental influences, macrophages are classified into two categories: classically activated macrophages (M1) and alternatively activated macrophages (M2) [9, 10]. M1 macrophages are characterized by the production of pro-inflammatory cytokines and then amplification of the inflammatory response, leading to organ dysfunction [11, 12], while the M2 phenotype function is associated with the secretion of high levels of anti-inflammatory cytokines and the reduction of inflammation [8]. Specifically, published data have suggested that the induction of macrophage transformation towards the M2 phenotype could be a potential therapeutic intervention for sepsis [13]. Thus, inducing macrophage M2 polarization and relieving excessive inflammation may be a way for MSCs to achieve therapeutic effects in sepsis.

Recently, studies have shown that paracrine activity plays an important role in the beneficial effects of MSCs [14]. MSC-secreted transforming growth factor beta (TGF-β) is an important factor that is associated with MSC immune modulatory function [15]. TGF-β is a well-known immunosuppressive factor that is involved in the inhibition of excessive inflammatory responses [16–18]. Furthermore, TGF-β induces macrophage M2 polarization, which can ameliorate macrophage-mediated inflammation [16, 19, 20]. Therefore, we speculated that the TGF-β secreted by MSCs can induce macrophage M2-like polarization to relieve excessive inflammation. However, the detailed mechanisms by which MSC-secreted TGF-β regulates macrophage M2-like polarization in an inflammatory environment have not been well elaborated.

The aim of this study was to determine the effects and mechanisms of MSC-secreted TGF-β on the LPS-stimulated macrophage polarization to the M2-like phenotype. We investigated the effects of MSC-secreted TGF-β on macrophage polarization and the inflammatory response in vitro by blocking TGF-β and by using TGF-β receptor (TGF-β R) inhibitors. Furthermore, we explored the mechanisms by which MSC-secreted TGF-β regulates macrophage M2-like polarization in an inflammatory environment.

## Materials and methods

### Ethics statement

The experiments were performed on male wild-type (WT) C57BL/6 mice (Experimental Animal Center, Nanjing, China), which were aged 10–12 weeks. Mice were maintained under specific pathogen-free conditions. Animal experiments performed conformed to the Guide for the Care and Use of Laboratory Animals. The Committee of Animal Care and Use of Southeast University approved this study.

### Isolation and identification of mouse peritoneal macrophages

Peritoneal macrophages were harvested from thioglycollate-injected mice. Three days prior to isolation, mice were injected intraperitoneally with 1.5 ml of 3% thioglycollate medium (Solarbio, China). The mice ascites was collected and centrifuged to obtain the cells, then the cells were seeded in the incomplete medium. After 3 h of incubation in 5% CO_2_, 37 °C, unattached cells were removed, then the adherent cells were cultured in complete medium. The phenotype of macrophages was identified by flow cytometry and immunofluorescence against F4/80.

### Cell culture

Murine MSCs were purchased from Cyagen Bioscience, Inc. (Guangzhou, China). The cells were cultured in Dulbecco’s modified Eagle’s medium/F12 (DMEM/F12; Wisent Biotechnology, Nanjing, China) containing 10% foetal bovine serum (FBS, Coring, Australia), 100 IU/ml penicillin, and 100 μg/ml streptomycin (Sigma-Aldrich, Munich, Germany) at 37 °C with 5% CO_2_. When cells reached 80–90% confluence, the adherent cells were trypsinized with 0.25% trypsin-EDTA (Invitrogen, American) and passaged into new flasks for further expansion. MSCs at passages 3–5 were used for the experiments. The murine-derived macrophage cell line RAW264.7 was purchased from Cells Resource Center of Shanghai Institutes for Biological Sciences, the Chinese Academy of Science. Mouse peritoneal macrophages and RAW264.7 cells were cultured in Dulbecco’s modified Eagle’s medium (DMEM; Wisent Biotechnology, Nanjing, China) containing 10% foetal bovine serum (FBS, Coring, Australia), 100 IU/ml penicillin, and 100 μg/ml streptomycin at 37 °C in a humidified atmosphere with 5% CO_2_.

### Co-culture protocol

Mouse peritoneal macrophages or RAW264.7 cells were plated at 2 × 10^6^ cells/well in the lower chamber of a six-transwell plate (Corning, USA) in the presence or absence of lipopolysaccharide (LPS; Sigma, Germany). MSCs were seeded in the upper chambers (0.4 μm pore size membrane) at 4 × 10^5^ cells/well. The medium volumes were 2 ml in the lower chamber and 2 ml in the upper chamber. After incubation, the culture supernatant and macrophages were collected for further experiments. The cells and supernatants were collected and stored at − 80 °C until further use.

### Reagent treatment

To evaluate the effect of LPS-induced RAW264.7 activation, the cells were treated with or without 500 ng/ml LPS for 12, 24, 48, and 72 h. The LPS-stimulated RAW264.7 cells were co-cultured with MSCs for 24, 48, and 72 h. The TGF-β R inhibitor LY2109761 (5 μM, Selleck, USA) or recombinant TGF-β (10 ng/ml, Sino Biological, China) were used to treat the cells for 48 h. Furthermore, equivalent volumes of complete medium were used as a negative control, and the Akt inhibitor GSK2141795 (30 μM, Selleck, USA) or the FoxO1 inhibitor AS1842856 (10 μM, Selleck) were used to inhibit the activation of the Akt/FoxO1 pathway in macrophages that were incubated with MSC-secreted TGF-β and LPS.

### Cell viability assay

The cytotoxic effect of LPS on RAW264.7 cells was analysed with a CCK-8 assay. RAW264.7 cells were seeded into 96-well plates (2000 cells in 100 μl medium) and incubated at 37 °C overnight and then treated with 10, 100, 500, or 1000 ng/ml LPS or without LPS for 24 h. Subsequently, 10 μl CCK8 (Beyotime, Chain) was added to each well and incubated for another 4 h. Finally, the absorbance was measured at 450 nm using a microplate reader.

### Enzyme-linked immunosorbent assay

For quantification, the expression levels of inflammatory cytokines: interleukin 6 (IL-6), interleukin 1β (IL-1β), interleukin 10 (IL-10). Following treatment, the supernatants were collected from each culture condition. ELISA was performed following the manufacturer’s instructions (R&D Systems, American).

### RAW264.7 cell phenotype analysis by flow cytometry

RAW264.7 cells were immune-labelled with antibodies against surface proteins. The antibodies that were used were anti-CD11b fluorescein isothiocyanate (FITC), anti-CD86 allophycocyanin (APC), and anti-CD206 phycoerythrin (PE) (BD Biosciences, San Diego, USA). RAW264.7 cells were collected, and an Fc receptor-blocking agent (Miltenyi Biotech, Germany) was used to block the Fc receptors for 5 min at 4 °C. RAW 264.7 cells were incubated with the antibodies in the dark at 4 °C for 30 min and then washed in PBS. The expression of CD86 and CD206 was calculated from the fluorescence intensity. All data were collected by flow cytometry (ACEA NovoCyte, China) using Novo Express (ACEA NovoCyte, China) and analysed using FlowJo software version X (Tree Star, USA).

### Immunocytochemistry

Mouse peritoneal macrophages were seeded on glass cover slips at a density of 2 × 10^6^ cells/well in six-well plates for 24 h. Cells were washed with PBS and fixed in 4% formaldehyde for 20 min. After washing with PBS, cells were permeabilized with 0.2% Triton X-100 and blocked with 1% BSA. Macrophages were incubated with a rabbit anti-F4/80 antibody (1:100, Cell Signaling, USA) diluted in PBS at 4 °C overnight. Next, cells were incubated with secondary antibodies (1:400, Yeasen, China) at 37 °C for 1 h. Finally, cell nuclei were counterstained with 4, 6-diamidino-2-phenylindole (DAPI). After a final wash, all images were observed with a fluorescence microscope.

### Quantitative real-time PCR analysis

Total RNA from RAW264.7 cells was extracted using TRIzol reagent (Life Technologies, USA). Reverse transcription of RNA and RT-PCR was performed by using Prime Script TM Trimester Mix (Takara, Japan) and SYBR Premix Ex TaqTM11 (Takara, Japan) with a Step One Plus RT-PCR system (Life Technologies, USA) according to the manufacturer’s instructions. β-actin was used as an endogenous control. Primers for RT-PCR were synthesized by Sangon Biotech (Shanghai, China). The primer sequences are as follows: mouse b-actin forward primer, 5′-GGGAAATCGTGCGTGAC-3′ and

reverse primer, 5′-AGGCTGGAAAAGAGCCT-3′;

mouse ARG-1 forward primer, 5′-AACACTCCCCTGACAACCA-3′ and

reverse primer, 5′-CATCACCTTGCCAATCCC-3′;

mouse iNOS forward primer, 5′- CAGCTGG GCTGTACAAACCTT-3′ and

reverse primer, 5′-CATTGGAAGTGAAGCGTT TCG-3′;

mouse IL-6 forward primer, 5′- CTTGGGACTGATGCTGGTGAC-3′ and

reverse primer, 5′- TTCTCATTTCCACGATTTCCCA-3′;

mouse IL-1β forward primer, 5′- TGTCTTGGCCGAGG ACTAAGG-3′ and

reverse primer, 5′-TGGGCTGGACTGTTTCTAATGC-3′;

and mouse IL-10 forward primer, 5′- GCTCT TACTGACTGGCATGAG-3′ and

reverse primer, 5′-CGCAGCTCTAGGAGCA TGTG-3′.

### Western blot analysis

Total proteins from macrophages were extracted using RIPA lysis buffer. The protein concentration of the cell lysates was determined with a BCA protein assay (Beyotime, China). Samples containing equal amounts of proteins were separated by 8% or 10% sodium dodecyl sulfate polyacrylamide gel electrophoresis and were transferred onto a PVDF membrane (Bio-Rad, American), followed by blocking with 5% BSA and incubation with specific primary antibodies against Akt, p-Akt, FoxO1, p-FoxO1, ARG-1, iNOS, and β-actin (Cell Signaling, USA, 1:1000) at 4 °C overnight. Following primary antibody incubation, membranes were washed and incubated with an HRP-conjugated secondary anti-rabbit or anti-mouse antibody (Beyotime, China, 1:5000) for 1 h at room temperature and visualized by using ECL detection kits (Beyotime, China).

### NO measurement

The level of NO was determined by measuring the quantity of nitrite in the supernatant by Griess reaction. Briefly, RAW264.7 cells were seeded in cell culture plate and incubated overnight. Thereafter, the cells were stimulated with LPS for 24 h, then cells were incubated in the presence of MSCs for different hours. To detect the nitrite concentration, the culture supernatant (50 μl) was mixed with Griess reagent 1(50 μl) and Griess reagent 11(50 μl) for 1 min. The nitrite concentration was determined by measuring the absorbance at 540 nm from the standard curve using sodium nitrite.

### Antibody microarray analysis

Protein phosphorylation detection was carried out with a Phospho Explorer Antibody Array (Catalog number AAM-APOSIG-1-8, Ray Biotech, USA), allowing for the examination of 17 phosphorylation sites (three replicates per antibody). Whole-cell lysates from RAW 264.7 cells that were starved for 24 h and treated with LPS, MSCs, or MSCs and a TGF-β R inhibitor (5 μM) were used for the antibody microarray analysis (*n* = 3), and the assay was performed according to the manufacturer’s protocol.

### Phagocytosis assay

RAW264.7 cells were stimulated with or without LPS for 24 h, and the LPS group was incubated in the presence of different drugs for 48 h. At the end of each treatment, RAW264.7 cells were incubated with fluorescein isothiocyanate (FITC)-OVA (100 μg/ml) at 37 °C for 4 h. The cells were washed twice with ice-cold PBS and analysed by flow cytometry, measuring the fluorescence intensity.

### Statistical analysis

All data were presented with GraphPad Prism 7.0 and are reported as the mean ± standard deviation. The statistical significance of the differences was compared using Student’s *t* test or one-way ANOVA analysis, followed by Bonferroni’s post hoc analysis. *P* < 0.05 was considered statistically significant.

## Results

### LPS increased pro-inflammatory cytokine levels and induced macrophages to the M1-like properties

**Fig. 1:**
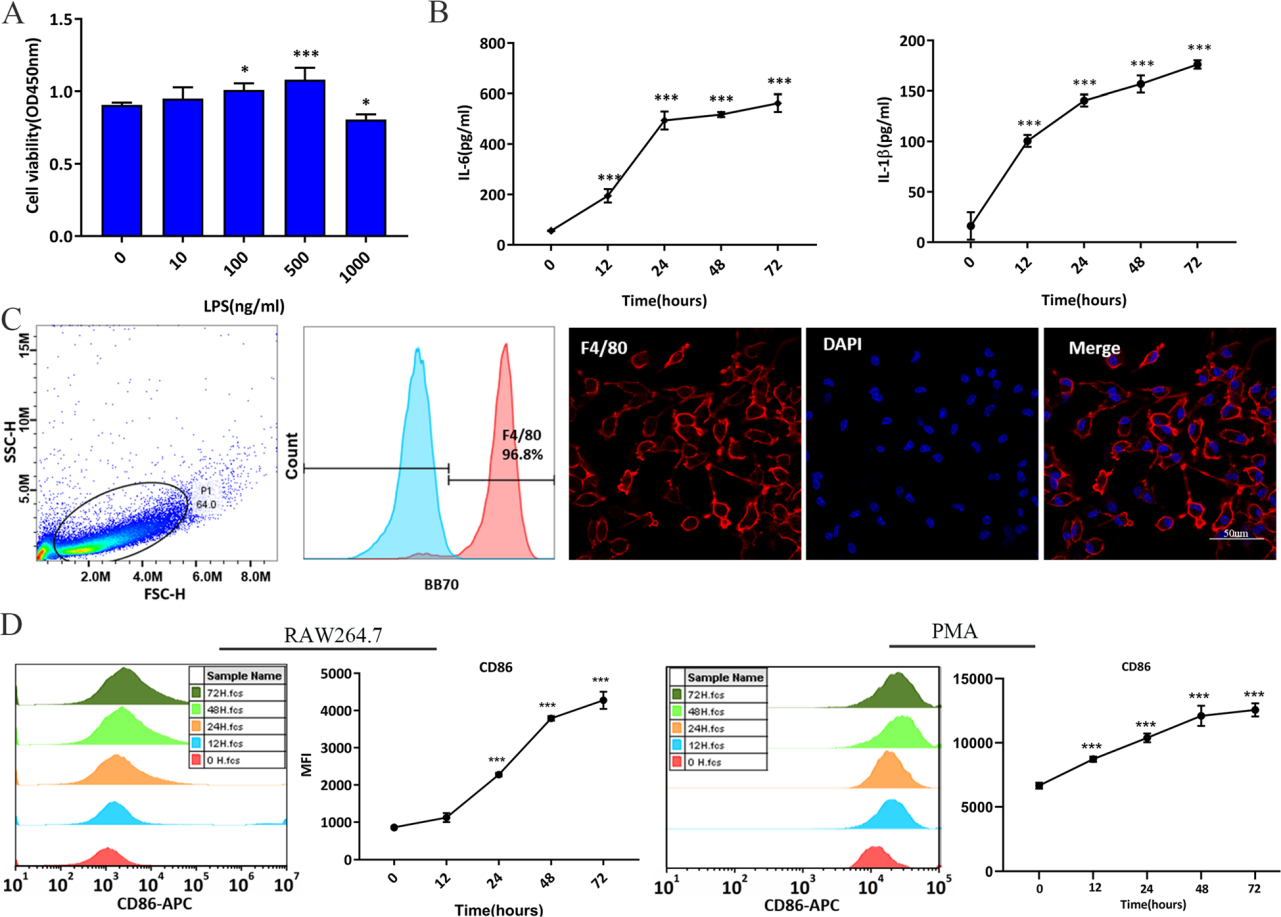
LPS-induced inflammatory reaction and M1-like properties macrophages. RAW264.7 cells were stimulated with or without LPS for 12, 24, 48, and 72 h. **a** The effect of different concentrations of LPS on cell viability in RAW264.7 cells at 24 h, as tested by CCK8. **b** The effect of LPS (500 ng/ml) on IL-6 and IL-1β production, as tested by ELISA. **c** Characteristics of mouse peritoneal macrophages by flow cytometry and immunofluorescence. **d** The effect of LPS (500 ng/ml) on MFI of CD86, as tested by flow cytometry. *p < 0.05 vs. control group; **p < 0.05 vs. control group; ***p < 0.001 vs. control group. Data show the mean ± SD (*n* = 3). APC, allophycocyanin; ELISA, enzyme-linked immunosorbent assay; IL-6: interleukin 6; IL-1β: interleukin 1β; LPS: lipopolysaccharide; MFI, mean fluorescence intensity; PMA: peritoneal macrophages

To verify the effect of LPS on RAW264.7 cells, we measured cell viability after LPS stimulation for 24 h. Compared to the control group, LPS at a concentration of 500 ng/ml significantly increased RAW264.7 cell viability but decreased the cell viability at a concentration of 1000 ng/ml (Fig. 1a). To examine the effect of LPS on RAW264.7 cell activation, we used LPS at 500 ng/ml to stimulate RAW264.7 cells for 12, 24, 48, and 72 h. Compared to the control group, LPS increased the levels of pro-inflammatory cytokines (IL-6 and IL-1β), and the concentration of these cytokines increased over time (Fig. 1b). In addition, CD86 was used as a marker of M1 macrophages, and flow cytometry analysis indicated that the mean fluorescence intensity (MFI) of CD86 followed similar trends to those reported above (Fig. 1d). After peritoneal macrophage culture, we confirmed that 96% of cells were F4/80-positive by flow cytometry and immunofluorescence (Fig. 1c). The MFI of CD86 in LPS-stimulated peritoneal macrophages accumulated in line with MFI of CD86 in LPS-stimulated RAW264.7 cells (Fig. 1d). Therefore, stimulation with 500 ng/ml LPS for 24 h was selected as the appropriate condition to induce a sufficient inflammation and M1-like property macrophages.

### MSCs suppressed the inflammatory reaction and converted the LPS-stimulated macrophages to an M2-like phenotype

**Fig. 2:**
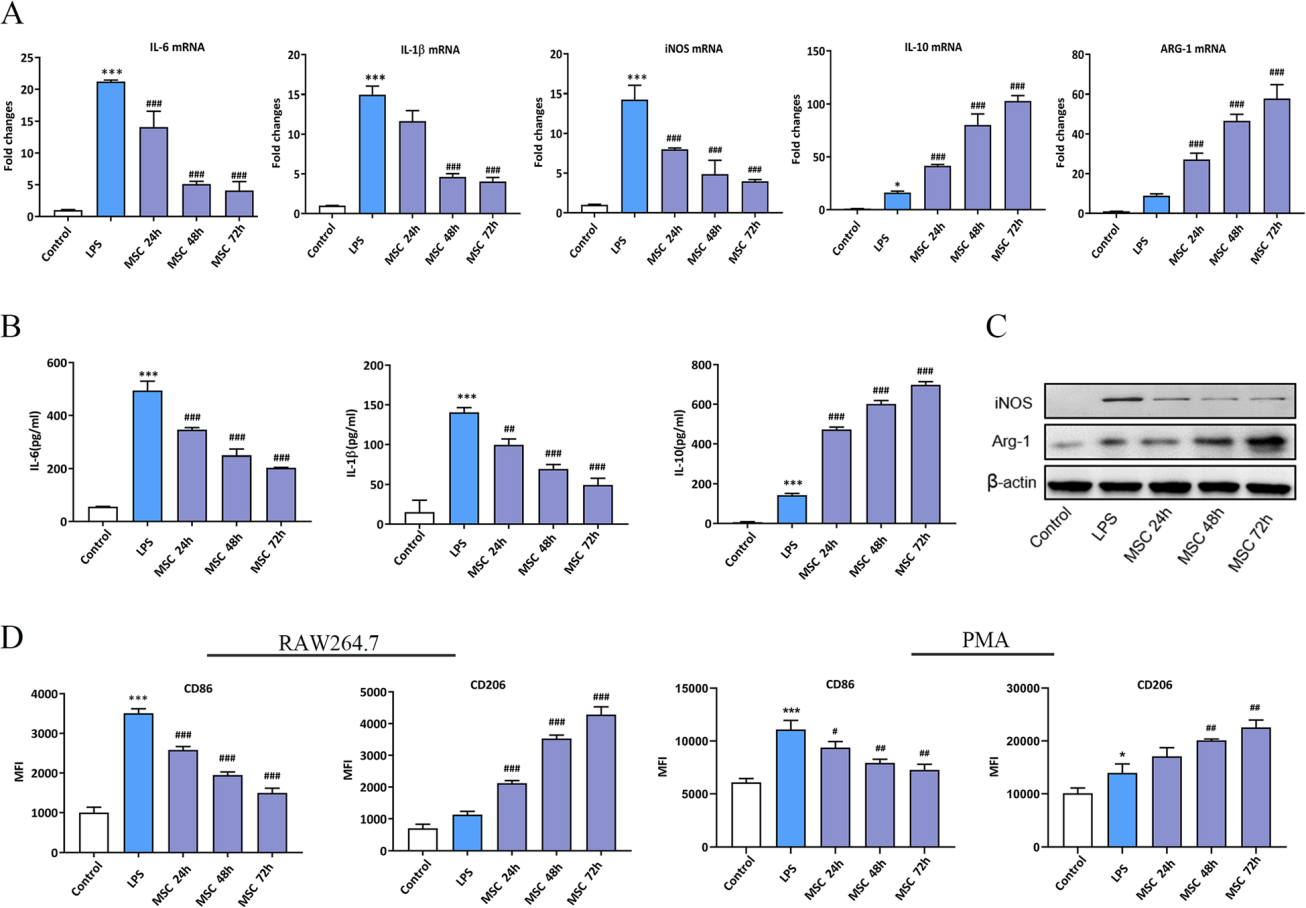
MSCs suppressed the inflammatory reaction and enhanced M2-like polarization in LPS-stimulated macrophages. **a** The effect of MSCs on inflammatory cytokine levels in RAW264.7 cells that were stimulated with LPS for 24, 48, and 72 h was tested by RT-PCR. **b**, **c** The effect of MSCs on inflammatory cytokine levels in RAW264.7 cells that were stimulated with LPS for 24, 48, and 72 h was tested by ELISA and WB. **d** The effect of MSCs on macrophage phenotypic marker levels in RAW264.7 cells and peritoneal macrophages that were stimulated with LPS for 24, 48, and 72 h was tested by flow cytometry. ***p < 0.001 vs. control group; #*p* < 0.05 vs. LPS group; ##*p* < 0.01 vs. LPS group; ###*p* < 0.001 vs. LPS group. (*n* = 3). ARG-1, arginase-1;ELISA, enzyme-linked immunosorbent assay; h, hours; iNOS, inducible nitric oxide synthase; IL-6, interleukin 6; IL-1β, interleukin 1β; IL-10, interleukin 10;LPS: lipopolysaccharide; MFI, mean fluorescence intensity; MSC: mesenchymal stem cell; PMA, peritoneal macrophages; RT-PCR, real-time PCR; TW, trans-well system; WB, Western blot

To evaluate the effects of MSC-secreted soluble factors on the macrophage phenotype, RAW264.7 cells were subjected to treatment with LPS for 24 h, then washed twice with PBS, followed by treatment with MSCs in a trans-well system (0.4 μm, Coring) for 24, 48, and 72 h. The mannose receptor (CD206) and arginase-1 (ARG-1) were used to indicate the M2-like phenotype, whereas CD86 and inducible nitric oxide synthase (iNOS) were used as markers of the LPS-stimulated macrophages. IL-6 and IL-1β are pro-inflammatory cytokines, while IL-10 is an anti-inflammatory cytokine. The results showed that at the mRNA level, compared to the controls, LPS obviously enhanced IL-6, IL-1β, and iNOS expression, while the production of IL-10 and ARG-1 slightly increased. MSCs diminished the pro-inflammatory cytokine levels in LPS-stimulated RAW264.7 cells in a time-dependent manner compared to those in the LPS-stimulated groups. However, the levels of IL-10 and ARG-1 significantly increased over time compared to those in the LPS-stimulated groups (Fig. 2a). The ELISA and Western blot (WB) results indicated that the trend in protein levels was consistent with the observed mRNA variation (Fig. 2b, c). We wonder whether the differences in iNOS/ARG1 protein lead to measurable differences in reactive nitrogen species production; the Griess assay results showed that compared to the controls, the concentration of NO was higher in LPS group, while MSCs diminished the level of NO in a time-dependent manner variation (Additional file 1: Figure S1). To further demonstrate that the MSCs induced macrophage phenotypic transformation, flow cytometry analysis confirmed that MSCs decreased the MFI of CD86 and increased the MFI of CD206 among LPS-stimulated RAW264.7 cells and peritoneal macrophages (Fig. 2d). These results suggested that MSCs reduced inflammation and skewed LPS-stimulated macrophages towards an M2-like polarization via a paracrine mechanism. In addition, MSCs induced LPS-stimulated macrophages to M2-like polarization in a time-dependent manner, and the effect of MSCs was more obviously at 48 h. Therefore, 48 h was taken as the time point for further study.

### MSCs induced the LPS-stimulated macrophages M2-like polarization via TGF-β

**Fig. 3:**
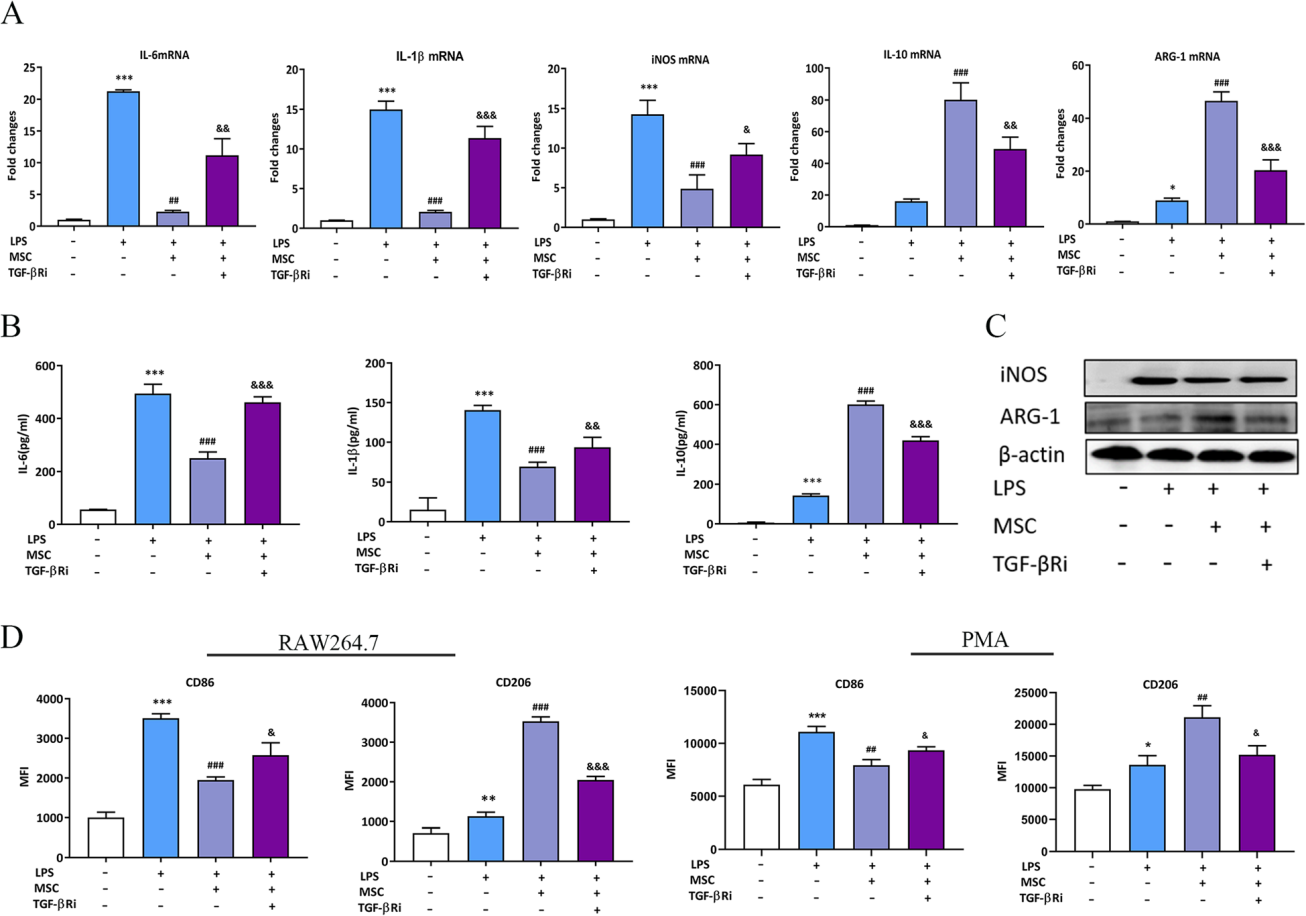
Paracrine TGF-β from MSCs suppressed inflammatory reaction and induced M2-like polarization in LPS-stimulated macrophages. **a** The effect of paracrine TGF-β from MSCs on inflammatory cytokine levels in LPS-stimulated RAW264.7 cells was tested by RT-PCR. **b**, **c** The effect of paracrine TGF-β from MSCs on inflammatory cytokine levels in LPS-stimulated RAW264.7 cells was tested by ELISA and WB. **d** The effect of paracrine TGF-β from MSCs on macrophage phenotypic marker levels in LPS-stimulated macrophages was tested by flow cytometry. *p < 0.05 vs. control group; **p < 0.01 vs. control group; ***p < 0.001 vs. control group; ##*p* < 0.01 vs. LPS group; ###*p* < 0.001 vs. LPS group; &*p* < 0.05 vs. MSC group; &&*p* < 0.01 vs. MSC group; &&&*p* < 0.001 vs. MSC group; (*n* = 3). ARG-1, arginase-1;ELISA, enzyme-linked immunosorbent assay; iNOS, inducible nitric oxide synthase; IL-6, interleukin 6; IL-1β, interleukin1β; IL-10, interleukin 10; LPS: lipopolysaccharide; MFI, mean fluorescence intensity; MSC: mesenchymal stem cell; PMA, peritoneal macrophages; RT-PCR, real time PCR; TGF-βRi, TGF-β receptor inhibitor; WB, Western blot

To evaluate the effect of MSC-secreted TGF-β on LPS-stimulated macrophage polarization, firstly, we measured TGF-β protein in supernatants from MSCs (Additional file 1: Figure S2). Then we utilized a co-culture model using LPS-stimulated RAW264.7 cells/peritoneal macrophages, and MSCs that were cultured in a trans-well system. The TGF-β in the system was blocked by a TGF-β receptor (TGF-β R) inhibitor, which was followed by the detection of cytokines and macrophage phenotypic markers. The results showed that MSC treatment reduced IL-6, IL-1β, and iNOS while increasing IL-10 and ARG-1 at both the mRNA and protein levels compared to those in the LPS groups; however, the effect of MSCs was significantly reduced by a TGF-β R inhibitor (Fig. 3a–c). We further investigated the macrophage surface markers with flow cytometry. The results showed that the MFI of CD86 was reduced and the MFI of CD206 was increased in the MSC treatment groups compared to those in the LPS-stimulated groups; however, the addition of the TGF-β R inhibitor in the system caused a reversal of the effects described above (Fig. 3d). Based on these results, we hypothesized that MSCs regulated the LPS-stimulated macrophage to M2-like polarization and reduced the inflammatory response by secreting TGF-β.

### Recombinant TGF-β mimics MSC-secreted TGF-β in LPS-stimulated RAW264.7 cells

**Fig. 4:**
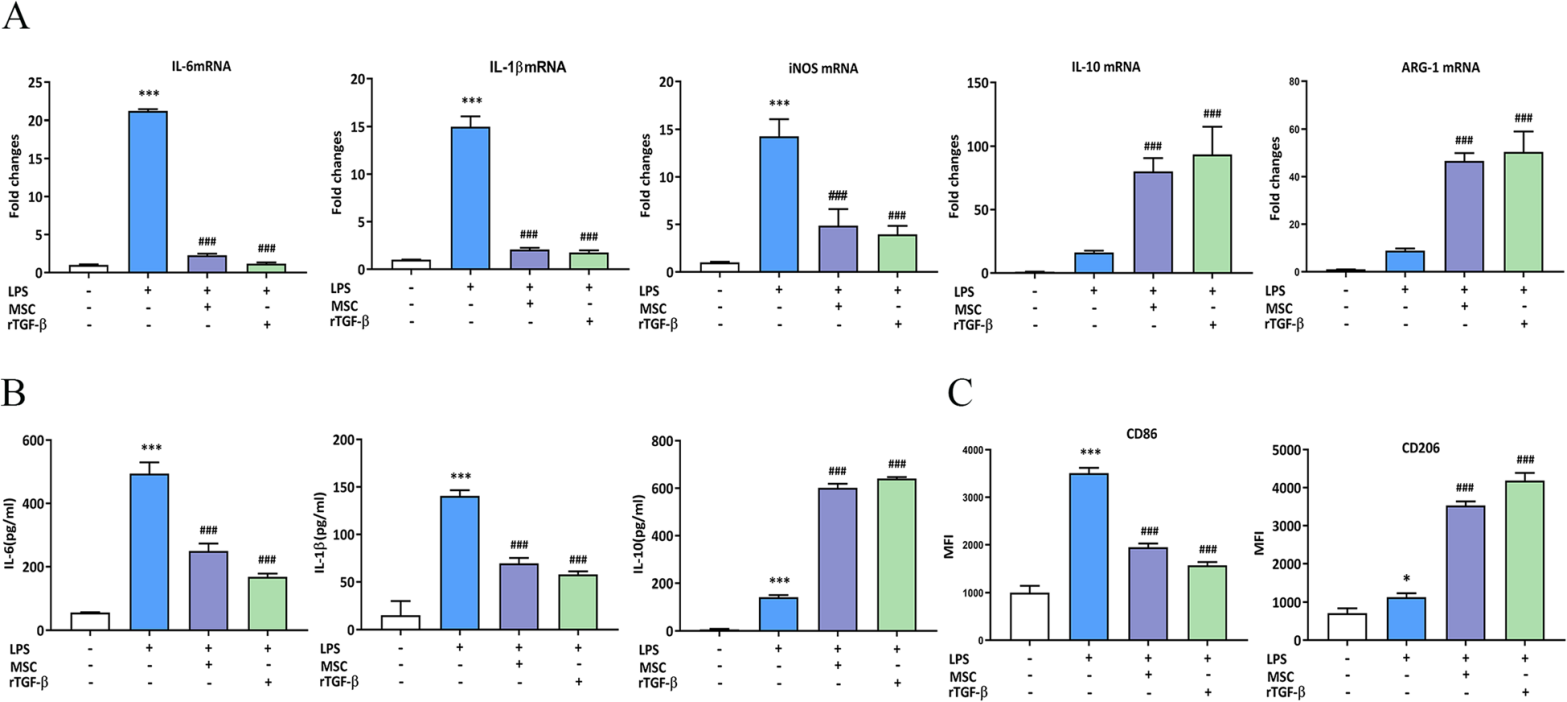
rTGF-β suppressed the inflammatory reaction and induced M2-liek polarization in LPS-stimulated RAW264.7 cells. **a** The effect of rTGF-β on inflammatory cytokine levels in LPS-stimulated RAW264.7 cells was tested by RT-PCR. **b** The effect of rTGF-β on inflammatory cytokine levels in LPS-stimulated RAW264.7 cells was tested by ELISA. **c** The effect of rTGF-β on macrophage phenotypic marker levels in LPS-stimulated RAW264.7 cells was tested by flow cytometry. *p < 0.05 vs. control group; **p < 0.01 vs. control group; ***p < 0.001 vs. control group; ###*p* < 0.001 vs. LPS group; &&&*p* < 0.001 vs. LPS group (*n* = 3). ARG-1, arginase-1; ELISA, enzyme-linked immunosorbent assay; iNOS, inducible nitric oxide synthase; IL-6, interleukin 6; IL-1β, interleukin 1β; IL-10, interleukin 10; LPS, lipopolysaccharide; MFI, mean fluorescence intensity; MSC, mesenchymal stem cell; rTGF-β, recombinant TGF-β; RT-PCR, real-time PCR

The effects of MSC-secreted TGF-β on LPS-stimulated RAW264.7 cells were confirmed, and we examined whether recombinant TGF-β (rTGF-β) had similar effects. To achieve the most robust effect of rTGF-β, a high concentration (10 ng/ml) of rTGF-β (Sino Biological, China) was used. RT-PCR and ELISA results showed that compared to the LPS groups, rTGF-β suppressed the levels of pro-inflammatory cytokines and increased the levels of anti-inflammatory cytokines in LPS-stimulated RAW264.7 cells, similar to the effect of paracrine TGF-β from MSCs (Fig. 4a, b). Likewise, rTGF-β inhibited CD86 expression and enhanced CD206 levels compared to those in the LPS groups, mimicking the effect of MSC-secreted TGF-β (Fig. 4c). These results suggest that rTGF-β regulated the macrophage phenotype in LPS-stimulated RAW264.7 cells.

### MSC-secreted TGF-β activated the Akt/FoxO1 signalling in macrophages that were stimulated with LPS

**Fig. 5:**
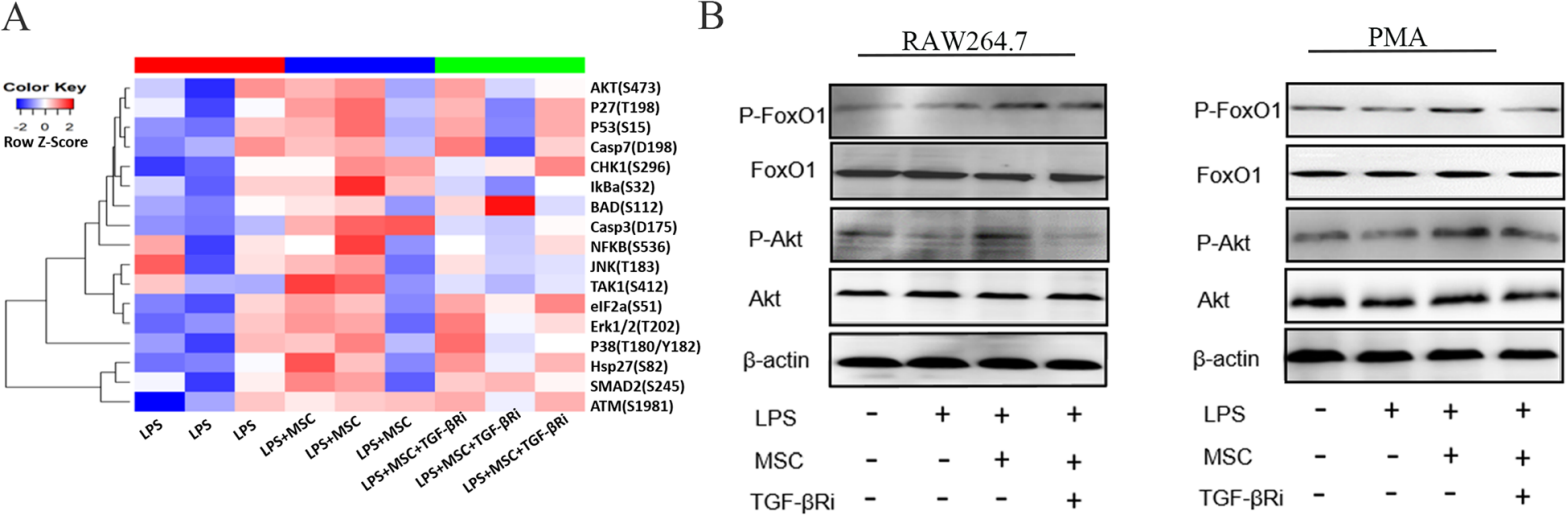
MSC-secreted TGF-β activated Akt/FoxO1 signalling in LPS-stimulated macrophages. **a** The protein expression levels were tested with antibody microarray analysis. **b** Western blot analysis revealed that there were significant increases in the phosphorylation levels of Akt and FoxO1 in the MSC groups compared to those in the LPS-stimulated groups, while TGF-β Ri decreased the phosphorylation levels of Akt and FoxO1 (*n* = 3). MSC, mesenchymal stem cell; PMA, peritoneal macrophages; TGF-β Ri, TGF-β receptor inhibitor

To explore the mechanisms by which MSC-secreted TGF-β induced LPS-stimulated macrophages to convert to an M2-like phenotype and reduced inflammation, we performed a phospho-antibody array on LPS-stimulated macrophages that were cultured with MSCs with or without a TGF-β R inhibitor in a trans-well system. The arrays contained 17 site-specific and phospho-specific antibodies. Among these antibodies, the highest signal was for phospho-Akt, which was increased by MSCs, but the increase was attenuated by co-treatment with a TGF-β R inhibitor (Fig. 5a). These changes, together with the literature reports, suggest that Akt induced macrophage M2 polarization [21, 22]. Therefore, we hypothesized that the Akt signalling pathways enhance the macrophage M2-like polarization induced by MSC-secreted TGF-β. We further validated the changes in the levels of these proteins in LPS-stimulated RAW264.7 cells/peritoneal macrophages using Western blotting (Fig. 5b). Since FoxO1 is a key downstream protein of Akt that mediates in immune regulation [23, 24], we examined the effects of MSC-secreted TGF-β on Akt/Foxo1 signalling in LPS-stimulated RAW264.7 cells/peritoneal macrophages. Compared to the LPS-stimulated group, MSC-secreted TGF-β increased the phosphorylation of Akt without changing total Akt protein levels in LPS-stimulated macrophages. Furthermore, the phosphorylation of Akt was gradually induced by MSC-secreted TGF-β at Ser 473, and the phosphorylation of FoxO1 was also increased, compared to those in the LPS-stimulated group. Co-treatment with a TGF-β R inhibitor partially reduced the MSC-secreted TGF-β-mediated Akt and FoxO1 phosphorylation (Fig. 5b). These results suggested that Akt/FoxO1 signalling is activated in the MSC-secreted TGF-β induced M1-like property macrophages to macrophages with an M2-like phenotype and is related to the reduction in inflammation.

### Akt signalling mediated the M2-like polarization in response to TGF-β in LPS-stimulated macrophages

**Fig. 6:**
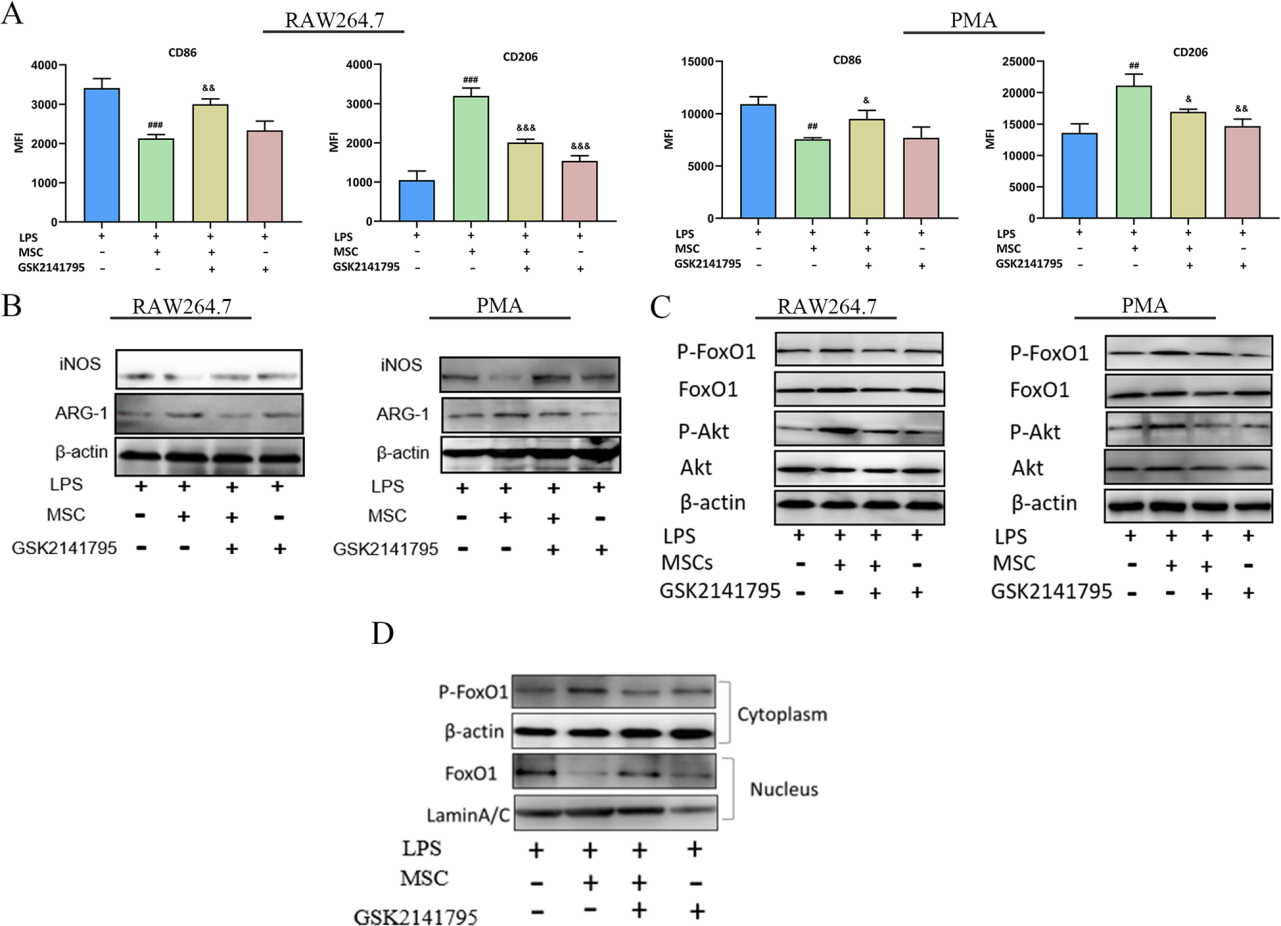
Effect of Akt inhibition on LPS-induced macrophages polarization in response to MSCs treatment. RAW264.7 cells were treated with or without GSK2141795 (30 μmol/l), followed by stimulation with LPS (500 ng/ml) or MSCs for 48 h. **a** The effect of GSK2141795 on the MSCs/LPS-induced RAW264.7 and peritoneal macrophages phenotype was tested by flow cytometry. **b** The effect of GSK2141795 on the MSCs/LPS-induced RAW264.7 and peritoneal macrophage phenotype was tested by WB. **c** Evaluation of the effect of MSCs on the Akt/FoxO1 signaling pathway with WB. **d** The effect of MSCs on FoxO1 translocated from the nucleus to the cytoplasm were tested by WB. The results are presented as the mean ± SD (*n* = 3). ###*p* < 0.001 vs. LPS group; &*p* < 0.05 vs. MSCs+LPS group; &&*p* < 0.01 vs. MSCs+LPS group; &&&*p* < 0.001 vs. MSCs+LPS group. GSK2141795, Akt inhibitor; LPS, lipopolysaccharide; MSCs, mesenchymal stem cells; PMA, peritoneal macrophages; rTGF-β, recombinant TGF-β; WB, Western blot

We next examined whether Akt signalling was involved in the TGF-β-mediated M2-like polarization in LPS-stimulated macrophages. RAW264.7 cells/peritoneal macrophages were pre-incubated with the Akt inhibitor GSK2141795 and then stimulated with LPS, followed by MSCs/rTGF-β in a trans-well system. Flow cytometry results showed that compared to the LPS-stimulated groups, MSCs/rTGF-β increased the MFI of CD206 and reduced the MFI of CD86 macrophages in LPS-stimulated groups, and GSK2141795 reversed those changes (Fig. 6a and Additional file 1: Figure S3a). Western blotting (WB) showed that compared to the MSCs/rTGF-β treatment groups, GSK2141795 increased the expression of iNOS and inhibited ARG-1, while GSK2141795 unregulated iNOS and ARG-1 in the LPS-stimulated group (Fig. 6b and Additional file 1: Figure S3b). To directly examine the mechanism of Akt in macrophage polarization, we detected the phosphorylation protein levels by performing WB analysis. Compared to the MSC treatment groups, after GSK2141795 stimulation, phosphorylated Akt (Ser473) and FoxO1 (Thr24) protein levels in LPS-stimulated macrophages were reversed (Fig. 6c). Importantly, the suppression of the Akt pathway decreased the p-FoxO1 level in the LPS-treated group compared to those in the MSC treatment groups (Fig. 6c), indicating that the Akt pathway may be involved in the regulation of the p-FoxO1/FoxO1 status in LPS-stimulated macrophages.

To investigate the potential regulation of FoxO1 localization and functions by MSC-secreted TGF-β, we used WB to detect the localization of FoxO1. As the Fig. 6d showed that FoxO1 was mostly localized in the nucleus in the LPS group, in response to MSC treatment, the level of p-FoxO1 was higher and the nuclear exclusion were enhanced. However, GSK2141795 inhibited the effects of MSCs secreted TGF-β on translocation of FoxO1. These results demonstrated that GSK2141795 prevented TGF-β-induced M2-like polarization in LPS-stimulated macrophages. In addition, FoxO1 activation and cytoplasmic retention were involved in TGF-β-induced M2-like polarization in LPS-stimulated macrophages.

### TGF-β regulated M2-like polarization in LPS-stimulated macrophages via FoxO1 and is Akt dependent

**Fig. 7:**
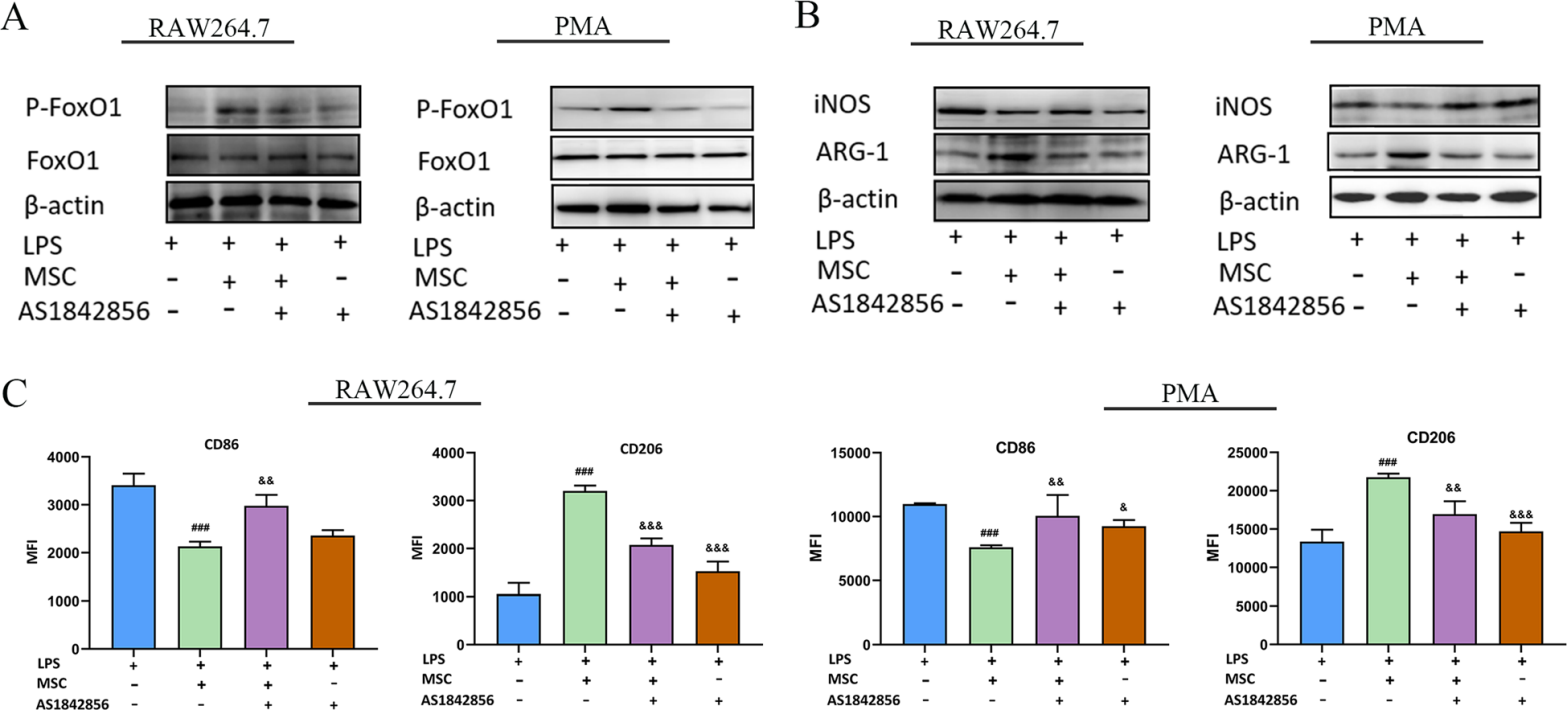
Effect of FoxO1 inhibitor on LPS-treated macrophage polarization in response to MSC treatment. Macrophages were treated with or without AS1842856 (10 nM), followed by stimulation with LPS (500 ng/ml) or MSCs for 48 h. **a** The effect of AS1842856 on FoxO1 was tested by WB. **b** The effect of AS1842856 on the LPS-treated RAW264.7 and peritoneal macrophages phenotype in response to MSCs treatment was tested by WB. **c** The effect of AS1842856 on the LPS-treated RAW264.7 and peritoneal macrophages phenotype in response to MSC treatment was tested by flow cytometry. The results are presented as the mean ± SD (*n* = 3). ###*p* < 0.001 vs. LPS group; &&*p* < 0.01 vs. MSCs+LPS group; &&&*p* < 0.001 vs. MSCs+LPS group. AS1842856, FoxO1 inhibitor; LPS, lipopolysaccharide; MSCs, mesenchymal stem cells; WB, Western blot

To further elucidate how FoxO1 regulates the TGF-β induced M2-like polarization in LPS-stimulated macrophages, we treated RAW264.7/peritoneal macrophages with the FoxO1 inhibitor AS1842856 and then treated the cells with LPS or MSCs/rTGF-β. AS1842856 is a powerful FoxO1 inhibitor that dramatically inhibited ARG-1 expression and promoted iNOS expression compared to that in the MSCs/rTGF-β groups. In addition, AS1842856 did not obviously change the levels of iNOS and ARG-1 in the LPS-stimulated group. (Fig. 7a, b and Additional file 1: Figure S4a-b). Compared to the MSCs/rTGF-β groups, AS1842856 also increased CD86 levels and reduced CD206 levels in LPS-stimulated macrophages, which reduced the response to MSCs/rTGF-β. (Fig. 7c and Additional file 1: Figure S4c). These results suggested that FoxO1 was dependent on MSCs/rTGF-β skewing the LPS-stimulated macrophages to M2-like polarization.

### Paracrine TGF-β from MSCs promoted macrophage phagocytosis

**Fig. 8:**
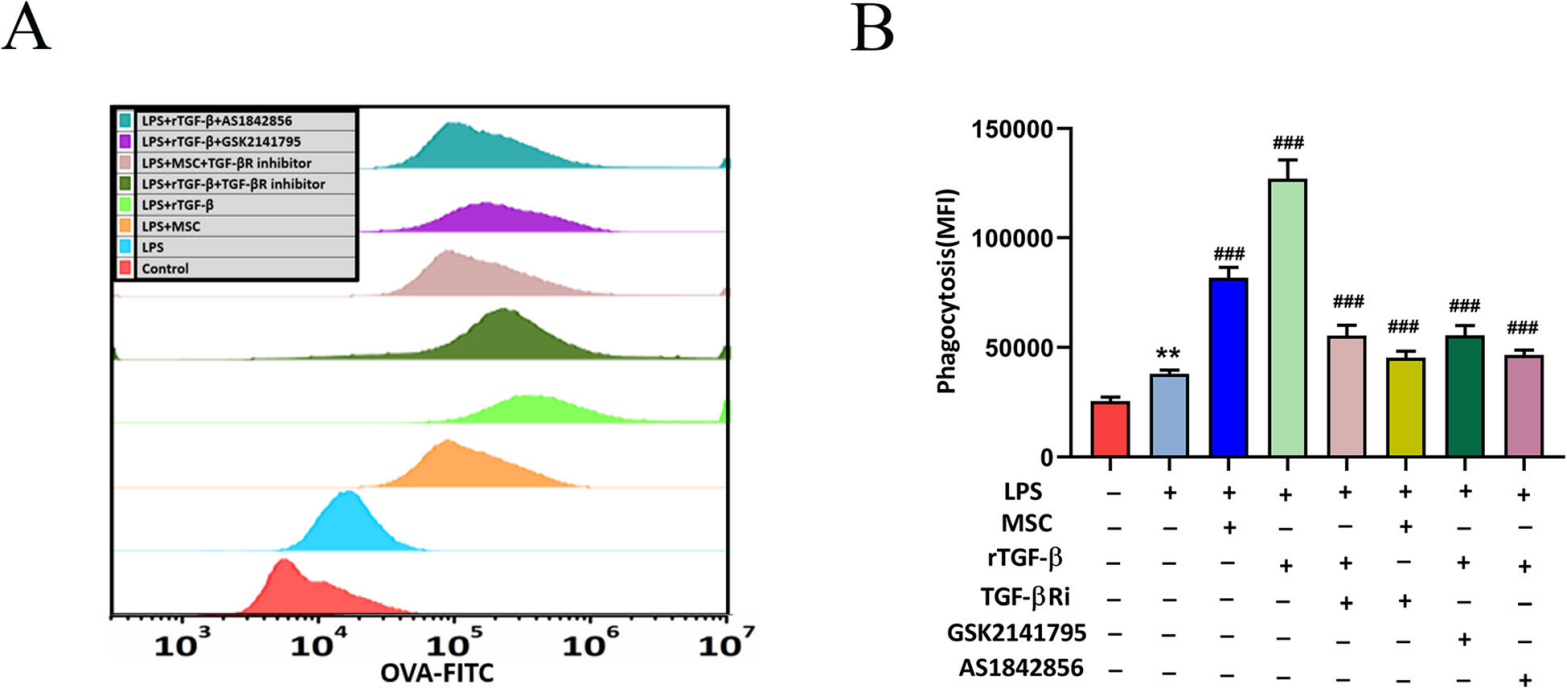
The effect of MSCs/rTGF-β on the phagocytic ability of RAW264.7 cells was tested by flow cytometry. **a**, **b** RAW264.7 cells were treated with or without AS1842856 (10 nM)/GSK2141795 (30 μmol/l), followed by stimulation with LPS (500 ng/ml) or rTGF-β (10 ng/ml)/MSCs for 48 h. The results are presented as the mean ± SD (*n* = 3). ***p < 0.001 vs. LPS group; ###*p* < 0.001 vs. LPS+rTGF-β group. AS1842856, FoxO1 inhibitor; FITC, fluorescein isothiocyanate; GSK2141795, Akt inhibitor; LPS, lipopolysaccharide; MSCs, mesenchymal stem cells; rTGF-β, recombinant TGF-β; TGF-βRi, TGF-β receptor inhibitor

We used OVA-FITC staining and flow cytometry to assess the phagocytosis of macrophages. LPS-stimulated RAW264.7 cells were treated with MSCs or rTGF-β (10 ng/ml) with LPS (500 ng/ml) in a trans-well system. We used a TGF-βR inhibitor to block the effects of MSCs or rTGF-β, and an Akt inhibitor and a FoxO1 inhibitor were used to inhibit Akt/FoxO1. The flow cytometry results indicated that LPS increased the phagocytic ability of RAW264.7 cells compared to that without LPS-stimulation group. In addition, upon co-culture with MSCs or rTGF-β, the RAW264.7 phagocytic ability further improved compared to that of LPS treatment alone. However, the TGF-βR inhibitor, AKT inhibitor, and FoxO1 inhibitor reversed these results (Fig. 8a, b). These results implied that TGF-β affects the phagocytic capacity of macrophages via the Akt/FoxO1 pathway.

## Discussion

Sepsis is characterized by the overwhelming activation of the immune response, which triggers an excessive inflammatory response and leads to high mortality [25]. Recently, it has become well known that MSCs are capable of reducing inflammation and improving survival by changing the macrophage phenotype during sepsis [6, 26, 27]. Particularly, growing evidence has shown that MSCs have beneficial effects as the result of a paracrine effect [28, 29]. However, the detailed mechanism through which MSCs regulate LPS-stimulated macrophage polarization and relieve excessive inflammation in sepsis remains unclear. In the present study, we found that MSCs skewed the M1-like phenotype towards the M2-like phenotype and significantly decreased the LPS-induced inflammatory reaction. This function of MSCs is primarily due to the paracrine effects of TGF-β. MSC-secreted TGF-β and rTGF-β had the same effect on the induction of macrophage polarization and the reduction of inflammation. Furthermore, our study demonstrated the involvement of the Akt/FoxO1 pathway in MSC-secreted TGF-β-mediated macrophage phenotype transformation, the inhibition of excessive inflammatory responses, and the enhancement of macrophage phagocytic ability. In addition, the inhibition of the Akt and FoxO1 pathway abolished the macrophage response to MSCs in an inflammatory environment. Taken together, these results showed that the TGF-β secreted by MSCs skewed the macrophage M1-like phenotype to the M2-like phenotype and reduced pro-inflammatory cytokine levels by activating the Akt/FoxO1 pathway in LPS-stimulated macrophages.

MSCs induce macrophage phenotypic changes, which in turn relieve inflammation and can be used as a therapeutic strategy in sepsis [30, 31]. Consistent with these findings, we found that the MSC treatment group had a higher level of M2 macrophage and lower inflammation than those obtained from the LPS-treated group. The expression of M2 markers and inflammatory cytokines among the total macrophages were measured using flow cytometry, RT-PCR and ELISA. At the mRNA and protein levels, compared to the LPS-stimulated group, pro-inflammatory cytokines, such as IL-1β and IL-6, were significantly reduced in the MSC-treated group, while the expression of the anti-inflammatory cytokine IL-10 was obviously increased. In addition, our results supported previous findings [30]. However, the mechanisms by which MSCs attenuate inflammation are still not completely understood.

Macrophage polarization plays a crucial role in sepsis [32, 33]. M1 macrophages are known to have pro-inflammatory effects, and CD86 and iNOS are markers of the M1 phenotype. The M2 phenotype has anti-inflammatory effects and is characterized by the specific expression of CD206 and ARG-1. In this study, we used LPS-stimulated macrophages to study the activation and induction of macrophage M1-like polarization. The results showed that the M1 markers and pro-inflammatory factors significantly increased after LPS stimulation. Furthermore, it demonstrated that MSCs skew the M1-like phenotype to the M2-like phenotype and inhibit the inflammatory response. Therefore, inducing LPS-stimulated macrophage M2-like polarization may be the mechanism by which MSCs prevent the over-activation of the inflammatory response.

MSCs can effectively regulate the inflammatory response via paracrine mechanisms. MSC secreted TGF-β is a crucial anti-inflammatory cytokine and is associated with immune regulation [16]. In our results, MSCs secreted TGF-β to induced LPS-stimulated macrophages to M2-like polarization. The secretion of TGF-β by MSC is affected by many factors. Current literature reported that the environment around MSCs is crucial to guide their paracrine activity [34]. MSCs perceive soluble cytokines and alter their paracrine activity, research found that TNF-α and IFN-γ could active MSCs and increase the level of TGF-β [35]. Furthermore, MSCs secreted TGF-β1 through auto-regulatory loop to upregulate the expression of TGF-β1 and 2 [36]. In addition, matrix-derived cues and mechanical forces also influence the paracrine ability of MSCs, mechanically loaded MSCs significantly increased the production of TGF-β [37]. Our results showed that in co-culture systems, MSC-secreted TGF-β decreased the levels of M1 markers, increased the expression of M2 macrophage markers, suppressed the over-activation of the inflammatory response; these effects could be restrained by TGF-β R inhibitors. Furthermore, rTGF-β alone sufficiently modulated LPS-stimulated macrophage effector function. In addition, Pender et al. recently showed that TGF-β improved the survival of rats with endotoxic shock by modulating peritoneal macrophage inflammatory mediator production, although the TGF-β used in this study was not released from stem cells [38]. Based on these findings and on our results, we proved that the TGF-β secreted by MSCs could skew macrophage M2-like polarization and attenuated LPS-induced inflammation.

Phagocytosis is a way for macrophages to clear bacteria and reduce inflammation during infections. M1 macrophages have a strong phagocytic ability, and whether TGF-β is secreted by MSCs affects the phagocytic ability of macrophages. Our data demonstrated that the TGF-β secreted by MSCs has the ability to increase macrophage phagocytosis; however, rTGF-β has stronger effects, which may be related to the concentration that is secreted by MSCs. Our study is consistent with previous reports, as the results showed that TGF-β is involved in macrophage phagocytosis [39]^.^ Increased macrophage phagocytosis may be involved in the reduction of excessive inflammatory responses.

One novel finding of this study was that MSC-secreted TGF-β regulated macrophage polarization, which was closely associated with the activation of the Akt/FoxO1 signalling pathway. Our study showed that the MSC-secreted TGF-β increased the expression of phosphorylated Akt, while a TGF-β R inhibitor decreased the expression of phosphorylated Akt in LPS-challenged macrophages. In addition, MSC-secreted TGF-β increased M2 markers and decreased M1 markers; however, GSK2141795, an inhibitor of Akt, reversed these changes. This indicated that Akt was involved in the regulation of the macrophage phenotype through MSC-secreted TGF-β. FoxO1, one of the key downstream targets of the Akt pathway, is an important nuclear transcription factor that regulates diverse cellular responses involving cell differentiation, cellular metabolism, and the inflammatory response [40, 41]. FoxO1 was reported to activate M1 expression directly, and FoxO1 dynamically regulates macrophage polarization through phosphorylation [42, 43]. FoxO1 is localized to the nucleus in its un-phosphorylated state, while Akt-mediated phosphorylation results in its nuclear export. The present study showed that p-FoxO1 (Thr24) in LPS-stimulated macrophages was increased by TGF-β, and FoxO1 was trans-located from the nucleus to the cytosol; meanwhile, the expression of M1 markers was reduced and the expression M2 markers were increased. Furthermore, both increased FoxO1 phosphorylation and nuclear exclusion induced by TGF-β were blocked by GSK2141795. In contrast, the effects of TGF-β were reduced upon Akt/FoxO1 inhibitor treatment. These findings highlighted that mechanisms involving Akt/FoxO1 phosphorylation and FoxO1 cellular translocation promote M2-like polarization and restrain the function of FoxO1 in promoting M1-like polarization. Additionally, MSC-secreted TGF-β inhibited excessive inflammatory activation via Akt/FoxO1 pathway activation.

Several limitations of this study should be noted. First, our study suggested that MSCs secreted TGF-β has a key role in the regulation LPS-stimulated macrophages M2-like polarization and relived excessive inflammation. However, the study was performed in vitro. Our future study research will in vivo to verify the role of MSC-secreted TGF-β. Second, during different stages of sepsis, the host’s immune response varies. Our study hypothesized that MSCs may be more effective in patients with hyper-inflammatory response/phase through reducing the excessive inflammatory response mediated by macrophages. However, the effect of MSCs in low inflammatory response/phase, especially in patients with severe immunosuppression remains unclear. It may induce immunosuppression in the late stage of sepsis which worth to confirm in the further study.

## Conclusion

In summary, this study demonstrated that treatment with paracrine TGF-β from MSCs skewed LPS-stimulated macrophage polarization to the M2-like phenotype, reduced inflammation, and increased phagocytosis. Additionally, the activation of the Akt/FoxO1 pathway contributed to the TGF-β regulatory effect in LPS-stimulated macrophages. These findings may improve our understanding of the complex mechanism of TGF-β-induced LPS-stimulated macrophage M2-like polarization and provide new insights for future sepsis therapeutic targets.

## Supplementary information

**Additional file 1: Figure S1.** MSCs suppressed the inflammatory reaction and enhanced M2-like polarization in LPS-stimulated macrophages. **Figure S2.** Paracrine TGF-β from MSCs suppressed inflammatory reaction and induced M2-like polarization in LPS-stimulated macrophages. **Figure S3.** Effect of Akt inhibition on LPS-induced RAW264.7 polarization in response to rTGF-β treatment. **Figure S4.** Effect of FoxO1 inhibitor on LPS-treated macrophages polarization in response to rTGF-β treatment.

## Data Availability

The data that support the findings of this study are available from the corresponding author upon reasonable request.

## Abbreviations

APC: Allophycocyanin
ARG-1: Arginase-1
ELISA: Enzyme-linked immunosorbent assay
FITC: Fluorescein isothiocyanate
IL-10: Interleukin 10
IL-1β: Interleukin 1β
IL-6: Interleukin 6
MFI: Mean fluorescence intensity
MSCs: Mesenchymal stem cells
PE: Phycoerythrin
PMA: Peritoneal macrophage
rTGF-β: Recombinant TGF-β
TGF-β R: TGF-β receptor
TGF-β: Transforming growth factor beta
WB: Western blot
