# Supplementary material for: MSC-secreted TGF-β regulates lipopolysaccharide-stimulated macrophage M2-like polarization via the Akt/FoxO1 pathway
Source: Stem Cell Res Ther. 2019 Nov 26;10:345. doi: 10.1186/s13287-019-1447-y (PMC6878630; doi:10.1186/s13287-019-1447-y)
Supplement: Supplementary file 1 — Additional file 1: Figure S1. MSCs suppressed the inflammatory reaction and enhanced M2-like polarization in LPS-stimulated macrophages. Figure S2. Paracrine TGF-β from MSCs suppressed inflammatory reaction and induced M2-like polarization in LPS-stimulated macrophages. Figure S3. Effect of Akt inhibition on LPS-induced RAW264.7 polarization in response to rTGF-β treatment. Figure S4. Effect of FoxO1 inhibitor on LPS-treated macrophages polarization in response to rTGF-β treatment. [file 13287_2019_1447_MOESM1_ESM.docx]

**
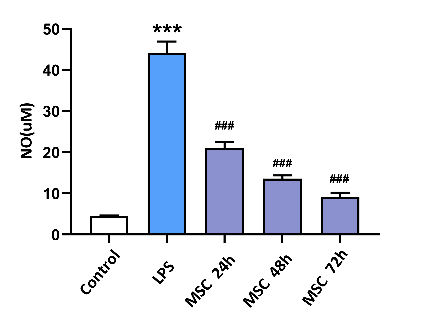
**

**Additional file 1: Figure S1** MSCs suppressed the inflammatory reaction and enhanced M2-like polarization in LPS-stimulated macrophages. The supernatant of treated groups were harvested and NO production was determined by Griess assay. *** p<0.001 vs. control group; ### p<0.001 vs. LPS group. (n=3). hr, hours; LPS: lipopolysaccharide; MSC: mesenchymal stem cell.


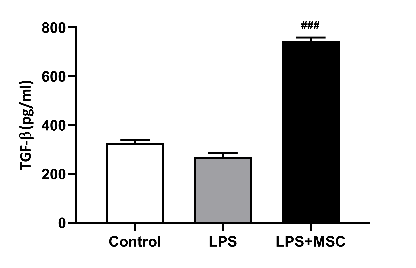


**Figure S2** Paracrine TGF-β from MSCs suppressed inflammatory reaction and induced M2-like polarization in LPS-stimulated macrophages. The TGF-β protein in supernatants from MSCs. ### p<0.001 vs. LPS group; (n=3). LPS: lipopolysaccharide；MSC: mesenchymal stem cell.


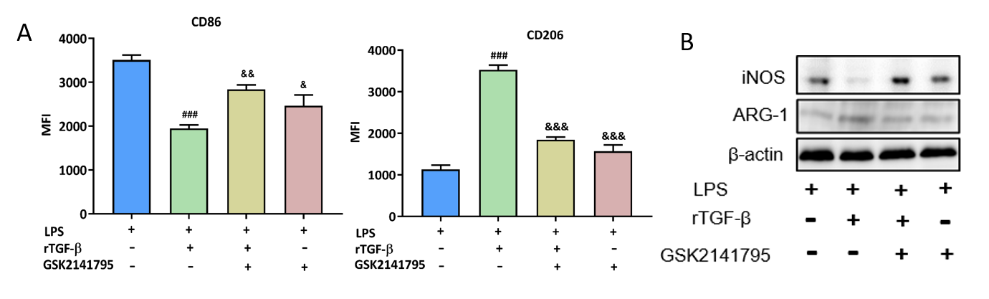


**Figure S3** Effect of Akt inhibition on LPS-induced RAW264.7 polarization in response to rTGF-β treatment. RAW264.7 cells were treated with or without GSK2141795 (30 µmol/L), followed by stimulation with LPS (500 ng/ml) or rTGF-β (10 ng/ml) for 48 hours. (a). The effect of GSK2141795 on the rTGF-β/LPS-induced RAW264.7 phenotype was tested by flow cytometry. (b).The effect of GSK2141795 on the rTGF-β/LPS-induced RAW264.7 phenotype was tested by WB. The results are presented as the mean ± SD (n =3). ###p<0.001 vs. LPS group; &p<0.05 vs. rTGF-β+LPS group; &&p<0.01 vs. rTGF-β+LPS group; &&&p<0.001 vs. rTGF-β+LPS group. GSK2141795, Akt inhibitor; LPS, lipopolysaccharide; MSCs, mesenchymal stem cells; rTGF-β, recombinant TGF-β; WB, western blot.


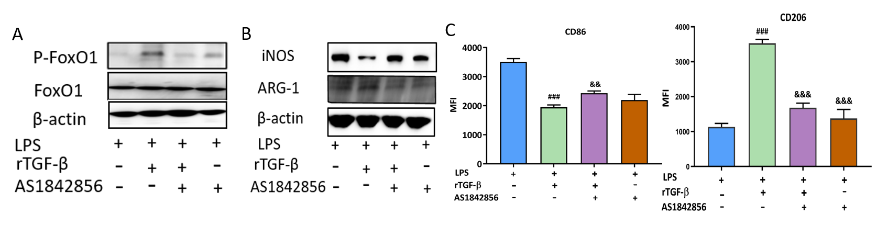


**Figure S4** Effect of FoxO1 inhibitor on LPS-treated macrophages polarization in response to rTGF-β treatment. Macrophages were treated with or without AS1842856 (10 nM), followed by stimulation with LPS (500 ng/ml) or rTGF-β (10 ng/ml) for 48 hours. (a). The effect of AS1842856 on FoxO1 was tested by WB. (b). The effect of AS1842856 on the LPS-treated macrophages phenotype in response to rTGF-β treatment was tested by WB. (c). The effect of AS1842856 on the LPS-treated macrophages phenotype in response to rTGF-β treatment was tested by flow cytometry. The results are presented as the mean ± SD (n = 3). ###p<0.001 vs. LPS group; &&p<0.01 vs. rTGF-β+LPS group; &&&p<0.001 vs. rTGF-β+LPS group. AS1842856: FoxO1 inhibitor; LPS, lipopolysaccharide; MSCs, mesenchymal stem cells; WB, western blot.
